# Inducible expression of cancer-testis antigens in human prostate cancer

**DOI:** 10.18632/oncotarget.12711

**Published:** 2016-10-17

**Authors:** Erika Heninger, Timothy E.G. Krueger, Stephanie M. Thiede, Jamie M. Sperger, Brianna L. Byers, Madison R. Kircher, David Kosoff, Bing Yang, David F. Jarrard, Douglas G. McNeel, Joshua M. Lang

**Affiliations:** ^1^ Department of Medicine, University of Wisconsin, Madison, Madison, WI 53705, USA; ^2^ Department of Urology, University of Wisconsin, Madison, Madison, WI 53705, USA; ^3^ University of Wisconsin Carbone Cancer Center, Madison, Madison, WI 53705, USA

**Keywords:** cancer testis antigen, prostate cancer, epigenetics, tumor immunotherapy, methylation

## Abstract

Immune tolerance to self-antigens can limit robust anti-tumor immune responses in the use of tumor vaccines. Expression of novel tumor associated antigens can improve immune recognition and lysis of tumor cells. The cancer-testis antigen (CTA) family of proteins has been hypothesized to be an ideal class of antigens due to tumor-restricted expression, a subset of which have been found to induce antibody responses in patients with prostate disease. We demonstrate that CTA expression is highly inducible in five different Prostate Cancer (PC) cell lines using a hypomethylating agent 5-Aza-2′-deoxycytidine (5AZA) and/or a histone deacetylase inhibitor LBH589. These CTAs include NY-ESO1, multiple members of the MAGE and SSX families and NY-SAR35. A subset of CTAs is synergistically induced by the combination of 5AZA and LBH589. We developed an *ex vivo* organ culture using human PC biopsies for *ex vivo* drug treatments to evaluate these agents in clinical samples. These assays found significant induction of SSX2 in 9/9 distinct patient samples and NY-SAR35 in 7/9 samples. Further, we identify expression of SSX2 in circulating tumor cells (CTC) from patients with advanced PC. These results indicate that epigenetic modifying agents can induce expression of a broad range of neoantigens in human PC and may serve as a useful adjunctive therapy with novel tumor vaccines and checkpoint inhibitors.

## INTRODUCTION

While often described as an indolent or treatable disease, prostate cancer (PC) remains the most common cancer and second leading cause of cancer-related death in US men (www.cdc.gov/uscs), as well as the cause of considerable morbidity. The sequential and concurrent use of hormonal and chemotherapies have been shown to improve survival for patients with advanced PC [1]. Resistance to these agents, unfortunately, is inevitable, and there is therefore a critical need to develop novel treatments [2]. Recent advances in immunotherapies, including anti-cancer vaccines (prostate cancer) [3] and checkpoint inhibitors (melanoma, lung and renal cancers) [4–6] have been shown to improve survival for patients with advanced disease. These therapies rely on the expression of tumor-associated antigens (TAAs) in the context of MHC class I for a CD8 T cell mediated anti-tumor immune response [7]. Expanding cancer immunotherapies to a broader range of patients could be achieved through novel target identification and vaccine development. Classical PC antigens currently in clinical trials include prostate specific antigen (PSA), prostatic acid phosphatase (PAP), androgen receptor (AR) and prostate-specific membrane antigen (PSMA) (NCT00583752, NCT00849121, NCT00694551).

Most notable is Sipuleucel-T, a dendritic cell vaccine with PAP as the target antigen, which improved survival in men with metastatic castration-resistant PC (CRPC) [8–10]. Advanced clinical trials are also evaluating the efficacy of agents targeting PSA (PROSTVAC-VF) [11] as well as PSMA (NCT02693860, NCT02552394) with promising results [12, 13]. However, the therapeutic potential in targeting these antigens may be limited for multiple reasons including 1) targeting “self antigens” to which the immune system may already be tolerized; 2) heterogeneous expression of target antigens in advanced disease; and 3) the ability of PC cells to escape immune detection via decreased expression of target antigens [14, 15].

An ideal tumor antigen is one to which the immune system is naïve and whose expression can be persistently induced in tumor cells. One class of TAAs that meet these criteria are the cancer testis antigens (CTAs). CTAs are a family of genes that are characterized by tissue-restricted expression to MHC class I deficient germline cells. CTAs are commonly expressed during early development and undergo epigenetic silencing in adult tissue, except in testis where they drive the gametogenic program. CTAs have also been identified in cancer and are expressed during the process of tumorigenesis and metastases via multiple mechanisms, including epigenetic alterations [16, 17]. To date, over 100 CTA families have been identified and 275 individual genes registered in the CTA Database (also referred to as CTPedia, www.cta.lncc.br) [18–20]. The role of CTAs in tumor progression is poorly understood but may include enhancement of the cell cycle, expression of mitotic machinery or suppression of apoptosis signaling cascades. Expression of these antigens in cancerous cells is quite variable. While some CTAs are especially prevalent in advanced or metastatic disease [21] such as SSX2 [22], significant heterogeneity in the expression profile of CTAs has been observed between different cancers including PC, tumor loci within the same host, or even within the same lesion [23–25].

The expression of CTAs in malignancy is heavily regulated by epigenetic alterations [17, 26, 27]. Cancerous cells demonstrate extensive hypermethylation of CTA gene promoter regions [28–31], and modulation of the epigenetic profile with the use of hypomethylating agents has been shown to strongly induce expression of CTAs in various types of cancer, including breast, colorectal, and ovarian *in vitro* [32] as well as *in vivo*. In a Phase II study in multiple myeloma, the hypomethylating agent azacitidine led to a significant increase of CTAs in the bone marrow compared to pre-treatment samples [33]. Interestingly, the considerable influence of epigenetic modifications on CTA expression appears to be unique to cancer cells. In studies using colon, skin, and prostate tissues, alterations in methylation status of CTA promoter sites was sufficient to induce expression of CTAs in tumor but not in normal epithelial cells [34–36].

The restricted expression pattern of CTAs in cancer cells and the ability to selectively augment expression with epigenetic modifying agents (EMAs) suggest that these antigens may be ideal immunotherapeutic targets for vaccine-based therapies. Prior work in a cohort of patients with prostatitis or prostate cancer identified antibodies against CTAs and were suggested to be potential autoimmune targets in prostate disease [37–40]. Novel anti-cancer vaccines targeting various CTAs have been developed across a wide variety of malignancies. These agents have demonstrated promising results in preclinical models and many have advanced to clinical trials. Early data suggest that these vaccines are overall well tolerated as well as efficacious. A seven peptide vaccine targeting CTAs in colon cancer resulted in disease stability or improvement in 60% of participants with colorectal cancer [41], and multiple vaccines in biliary cancer have been associated with stable disease in 50% or more of trial participants [42, 43], all with limited toxicities in Phase I trials. Furthermore, in Phase II studies, evaluation of a prime and boost vaccination strategy targeting the CTA, NY-ESO1, in late-stage melanoma and ovarian cancer demonstrated clinically meaningful responses [44], and in patients with advanced esophageal cancer, immune responses induced by a CTA vaccine resulted in improved survival [45]. These new therapeutic vaccines could be of use in multiple disease types if the target of interest was expressed.

The goal of this study was to determine the expression pattern and inducibility of CTAs in human PC. Expression patterns of 29 potentially immunologically relevant CTAs were evaluated in 5 PC cell lines of which 21 CTAs were induced following treatment with EMAs. This expression pattern varied across different CTAs and was at least partially related to the androgen sensitivity of these cell lines. Human PC biopsies showed almost no expression of SSX2 or NY-SAR35 at baseline but could be significantly induced following treatment with EMAs. Gene expression analysis of CTAs in circulating tumor cells further identified a subset of patients with metastatic PC that could benefit from vaccines targeting CTAs. These results identify a translational paradigm in which combining EMAs with CTA-targeted vaccines in patients with PC can enhance immune-mediated tumor lysis.

## RESULTS

### Expression of CTAs *in vitro*

**Figure 1:**
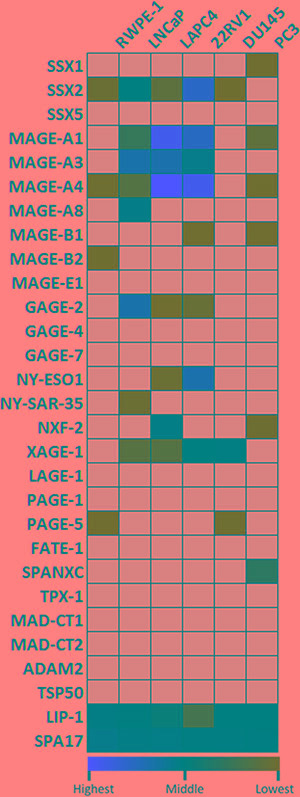
Quantitative analysis of baseline expression of cancer/testis antigen mRNA in 5 PC cell lines and in the RWPE-1 normal epithelial cell line RNA was evaluated by qRT-PCR for expression relative to an internal control transcript (P0). qRT-PCR was performed using primers specific for each gene, conducted in triplicate, and repeated in an independent experiment. Expression relative to P0 was color scaled from red (highest) to black (middle) to green (lowest). Grey indicates that no relevant expression was detected.

We evaluated 29 CTAs that had previously been identified in patients with prostate disease using antibody screening methodology and may be immunologically relevant in prostate cancer (Table 1) [37–40, 46, 47]. We tested baseline expression of these 29 CTAs in three AR-expressing PC cell lines (22rv1, LNCaP, LAPC4), two androgen-independent cell lines (PC3, DU145) and a benign prostate epithelial cell line (RWPE-1) (Figure 1). Among the 29 CTAs selected for this study, there were 22 X-linked and 7 non-X linked antigens. The relative expression of CTA mRNA to internal control transcript RPLP0 (P0) showed considerable variation across cell lines, in both expression levels and the range of CTAs detected. From the SSX family, SSX2 was detected in all PC cell lines except PC3. Notably, AR-expressing PC cell lines, particularly 22rv1 and LNCaP expressed higher levels of SSX2 than the two androgen-independent lines chosen for this study (Figure 1 and Figure S2). The MAGE-A family members were detected across several cell lines with MAGE-A3 and MAGE-A4 highly expressed in AR-expressing cells lines, not detectable in DU145, and detectable in PC3 cells at lower levels. Among the non-X linked CTAs, both LIP1 and SPA17 were expressed across all tested cell lines. Taken together, AR-expressing cell lines expressed higher levels and higher diversity of CTAs compared to androgen-independent PC cell lines and normal epithelial cells. Additionally, eleven of the CTAs chosen in this study had detectable expression in at least one PC cell line but not in RWPE-1 (SSX1, MAGE-A1, MAGE-A2, MAGE-A8, MAGE-B1, GAGE-2, NY-ESO1, NY-SAR35, NXF-2, XAGE-1 and SPANXC), and twelve were undetectable in all cell lines tested (MAGE-E1, GAGE-4, GAGE-7, PAGE-1, LAGE-1, SSX5, FATE-1, TPX-1, MAD-CT1, MAD-CT2, ADAM2 and TSP50).

**Table 1:** Names, alias, CT Identifiers (http://www.cta.lncc.br), GeneBankIDs and primers are listed for the 29 CTAs selected for this study

Gene (alias)	GenBankID	CT identifier	Forward Primer	Reverse Primer
MAGE-A1	NM_004988	CT1.1	CACCTCCTCCTCCTCTCCTC	TCTCCAGCATTTCTCGCCTTT
MAGE-A3	BC016803	CT1.3	GCAAAGCTTCCAGTTCCTTG	AAATGTTGGGTGAGCAGCTT
MAGE-A4	BC017723	CT1.4	GCAAGTATCGAGCCAAGGAG	TCCCAGATTTCCTCCTCAGA
MAGE-A8	BE387798	CT1.8	AGAAGTGGACCCCTTTGTCC	GGATCACTATTGGGCACCTG
MAGE-B1	BE897525	CT3.1	TGCTGCAGCTGTGTCATGTA	TGGCCACTAGGGTTGTCTTC
MAGE-B2	BC026071	CT3.2	CTTCAAGCTCTCCTGCTGCT	GGAAGTGCTCCCTGAACCTT
GAGE-2	BC069397	CT4.2	GCCTAGACCAAGACGCTACG	CCTTCTTCAGGCGTTTTCAC
GAGE-4	BC069470	CT4.4	GCCTAGACCAAGGCGCTAT	CCTTCTTCAGGCGTTTTCAC
GAGE-7	NM_021123	CT4.7	GGAATTCATGAGTTGGCGAGGAAGATCGACC	CCGCTCGAGTTAACACTGTGATTGCTTTTCACC
SSX-1	BC001003	CT5.1	CTAAAGCATCAGAGAAGAGAAGC	AGATCTCTTATTAATCTTCTCAGAAA
SSX-2	BC007343	CT5.2	GGTGCTCAAATACCAGAGAAG	GGTGCTCAAATACCAGAGAAG
SSX-5	BC016640.2	CT5 family*	GTTCTCAAATACCAGAGAAGATG	CTCTGCTGGCTTCTCGGGCG
NY-ESO-1	AJ003149	CT6.1	GCTTCAGGGCTGAATGGAT	AAAAACACGGGCAGAAAGC
LAGE-1	BC002833	CT6.2.b	TTCTGCGCAGGATGGAAG	AAAAACACGGGCAGAAAGC
MAGE-E1 (MAGED4)	BC081566	CT7.1	AGAGCATCACAGCCCTCATT	TCAGGTGGATCCCAAACTTC
SPANXC	BC054023	CT11.3	AATGGACAAACAATCCAGTGC	CATGAATTCCTCCTCCTCCA
XAGE-1	BC009538	CT12.1.a	GTATCCGAGTCCCAGAAGCA	GATTTATCCCCGGTGTTTGA
ADAM2	BC064547	CT15	TGCACCCCAGAACCATAAGT	CTCTCCTGCTTCCAGCTTTG
PAGE-5	BC009230	CT16.1	CCCAATCCTCAGAAAGAGGAA	TTCACCTGCTTCCAGCACTT
PAGE-1	BC010897	CT16.3	TCCAGAGGAAGAGGAGATGG	CTTAGCACGCTCCGGATTAG
LIP-1	BC023635	CT17	CAGACCGTTAAGCTCCTTGC	GGTGGCATATTCTCCACCTC
TSP-50	BC037775	CT20	TGAAACCCTGCAAGGAAAAC	GCACTGAGGGAGTCCACAAT
SPA17	BC023457	CT22	ACGCGAGATTCTGAGAGAGC	CAGCTTGGATTTTGACAGCA
TPX-1 (CRISP2)	BC022011	CT36	AGAGGACCGCAAAACCAGTA	TTCCTTGTTGGTACGGGGTA
NY-SAR-35	BC034320	CT37	AAAGCGAAGGGGAGGAATAG	GGGCAGGATATGTCCATTTG
NXF-2	BC015020	CT39	TGAAACCCTGCAAGGAAAAC	GCACTGAGGGAGTCCACAAT
FATE-1	BC022064	CT43	GAAATGTCCCTGGCAGAAGA	AATGGAAACGTATGCCTTGG
MAD-CT1	NM_002762	CT94.2	AGGTGTACAGGCAGCAGTTG	TCTGCATGTTCTCTTCCTGGT
MAD-CT2	AK097414	*	ACCCTGAGACAACCACTTCG	ACCTGTCTGCCTTCTTGTGC

* CTAs not assigned a CT Identifier number, yet.

### EMAs induce CTA expression *in vitro*

**Figure 2:**
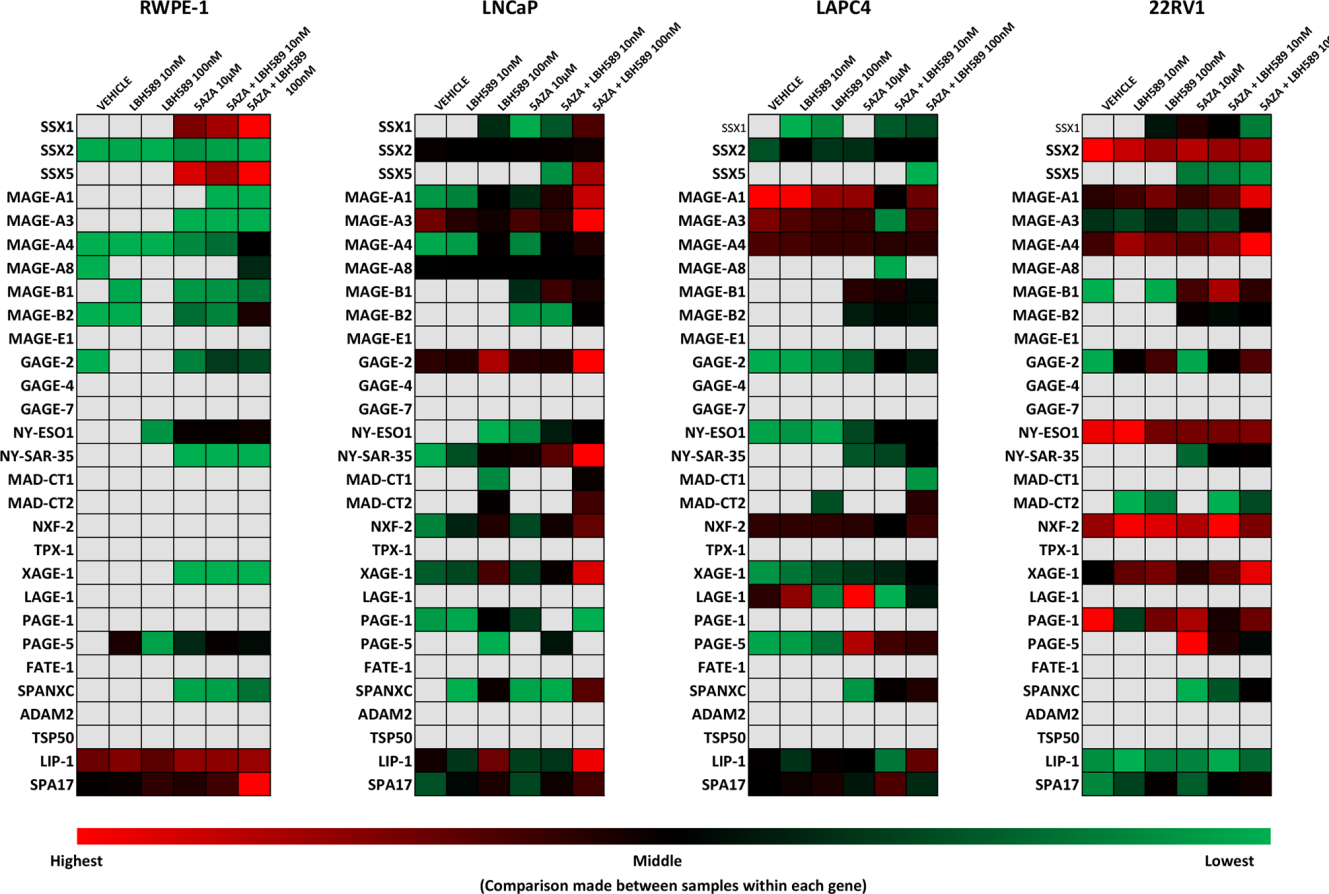
Quantitative analysis of relative expression of CTA mRNA in androgen-receptor expressing PC cell lines and in RWPE-1 normal epithelial cells in response to EMA treatment RNA was evaluated by qRT-PCR for expression relative to an internal control transcript (P0) following EMA treatment of RWPE-1, LNCaP, LAPC4, and 22rv1 cells. qRT-PCR was performed using primers specific for each gene, conducted in triplicate, and repeated in an independent experiment. Expression relative to P0 was color scaled from red (highest) to black (middle; 50th percentile) to green (lowest) for each CTA separately, across all cell lines, thus similar colors between different rows cannot be directly compared. Grey indicates that no relevant expression was detected.

**Figure 3:**
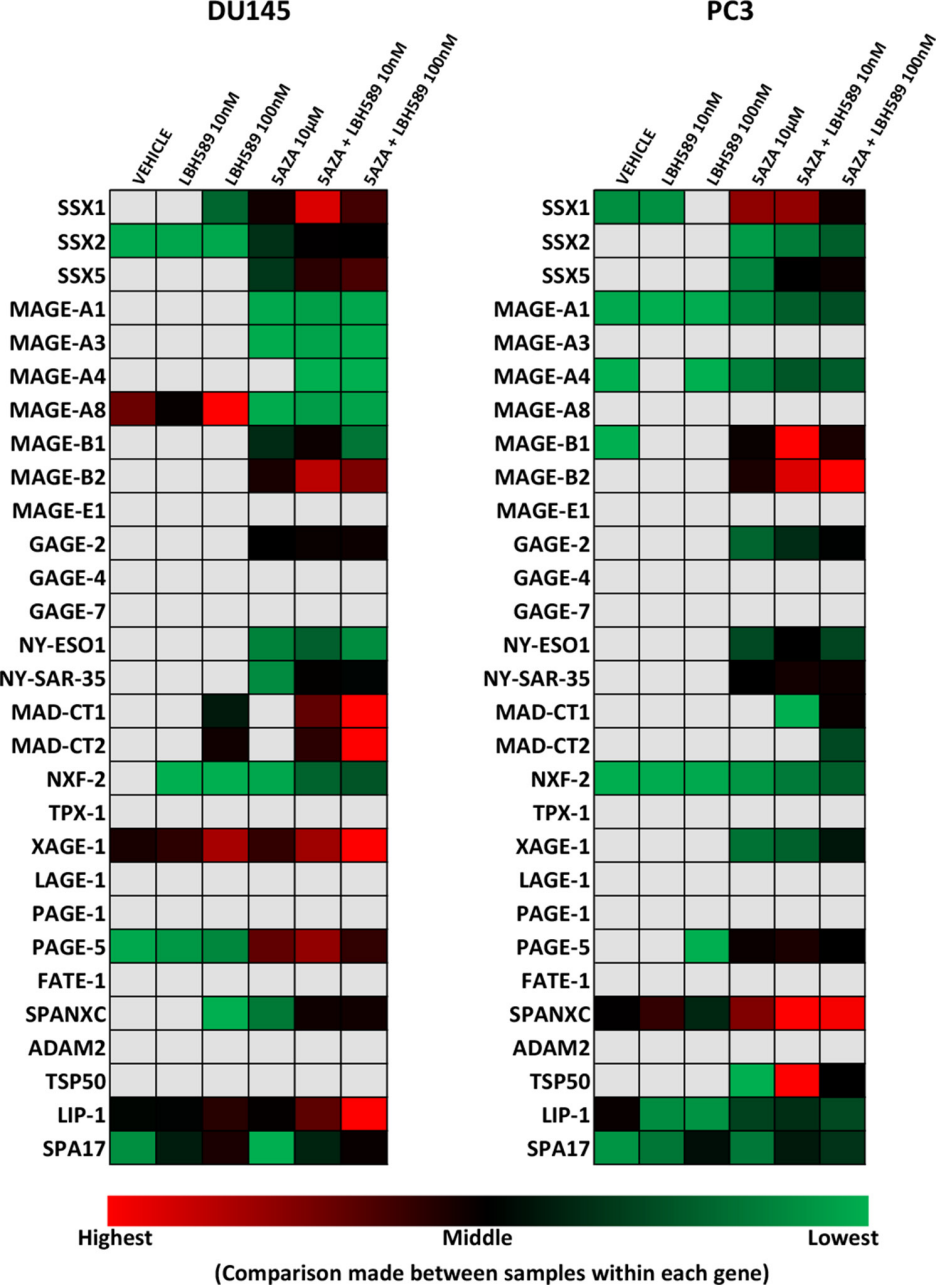
Quantitative analysis of relative expression of CTA mRNA in androgen-independent PC cell lines treated with EMAs RNA was evaluated by qRT-PCR for expression relative to an internal control transcript (P0) following EMA treatment of DU-145 and PC3 cells. qRT-PCR was performed using primers specific for each gene, conducted in triplicate, and repeated in an independent experiment. Expression relative to P0 was color scaled from red (highest) to black (middle; 50th percentile) to green (lowest) for each CTA separately, across all cell lines, thus similar colors between different rows cannot be directly compared. Grey indicates that no relevant expression was detected.

Epigenetic modification has been previously shown to be a key factor in regulation of CTA expression [17, 26, 27, 37]. We tested if EMAs shown to induce CTA expression in other tumor types could have a similar effect in PC. We utilized the hypomethylating agent 5AZA and/or the histone deacetylase (HDAC) inhibitor LBH589 to induce CTA expression in PC cell lines (Figures 2 and 3). Data is also shown in bar graphs in Supplementary Figures S1, S2, S3, S4 and S5 depicting statistical significance between treatment groups.

In both androgen-independent cell lines (DU145 and PC3) (Figure 3), combinatorial treatment significantly increased SSX2 expression relative to baseline. Although there was some limited induction of SSX2 in LNCaP and LAPC4 cells, epigenetic treatment was much less effective in AR-expressing cell lines (Figure 2). Both SSX1 and SSX5 were inducible (Figure S2. *top row, left panel* and *bottom row, left panel*, respectively) by combination treatments across all cell lines, though at much lower expression levels than SSX2. Similarly, a significant increase of protein expression of the SSX family was also detected in PC3, DU145 and in LAPC4 cells following combinatorial treatment (Figure S2, *bottom right panel*).

Combinatorial treatments resulted in a significant induction of most MAGE-A family members in most cell lines tested (Figures 2 and 3, shown as bar graphs in Supplementary Figure S1). MAGE-A1 and MAGE-A4 were both induced across all cell lines with the exception of LAPC4 cells. Additionally, they were both highly induced in 22rv1 cells on top of very high baseline expression, resulting in the highest expression levels detected after epigenetic treatments for all CTAs tested. However, MAGE-A1 expression levels remained near the detection limit in RWPE-1 cells after all treatments. While MAGE-A3 was induced in DU145 by both 5AZA alone and combination treatments, it remained undetectable in PC3 cells, and the AR-expressing cells lines showed limited induction of this tumor antigen. We also evaluated other members of the MAGE family (Figures 2, 3, Supplementary Figure S1 and S3). MAGE-A8 had relevant expression only in LNCaP cells, and expression was not affected by EMAs. Although both MAGE-B1 and MAGE-B2 baseline expression stayed close to or under detection limit across all the analyzed cells lines, both 5AZA and combination treatments rescued MAGE-B1 and MAGE-B2 in all the PC cell lines while RWPE-1 cells were only induced by high dose combination treatment. MAGE-B2 expression was very highly induced by combinatorial treatments in the androgen-independent cell lines.

While NY-SAR35 expression was only detected in LNCaP cells at baseline and at a relatively low level, combinatorial treatment highly induced its expression across all PC cell lines but only moderately in the benign prostate cell line. Combinatorial treatments also resulted in a significant increase of NY-ESO1 expression across all cell lines with the exception of 22rv1, which had the highest baseline expression of NY-ESO1 prior to treatment. Additionally, LBH589 alone resulted in a significant increase of NY-ESO1 expression over vehicle in 22rv1 cells. Of the other CTAs studied, GAGE2, PAGE5, XAGE1, NXF2, SPANXC, and MADCT2 were all notable for being significantly induced across all PC cell lines tested. In RWPE1 cells, however, XAGE1, NXF2, SPANXC and MADCT2 expression all remained near or below the detection limit.

### Epigenetic treatment induces CTA expression in human PC *ex vivo*

**Figure 4:**
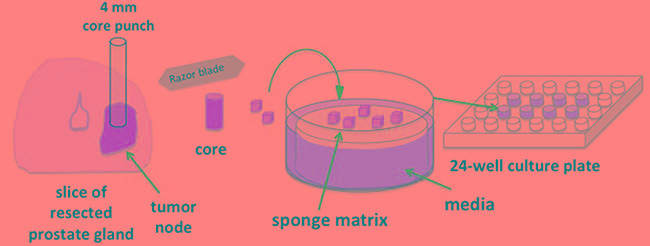
*Ex vivo* human prostate tissue drug culture A novel preclinical model developed to test the effect of EMA agents on prostate tumor tissue collected from patients undergoing radical prostatectomy. Resected prostate gland slices are subject to gross pathology examination. 4 mm cores are punched from visible tumor nodes and tissue is sliced into ~1 mm^3^ pieces. Surgical gelatin sponge is soaked in supplemented Ham's media and placed in 24-well tissue cultures wells. Tissue slices are carefully layered on the surface of saturated sponge matrix. The matrix is fully saturated by but is not submerged in media.

**Figure 5:**
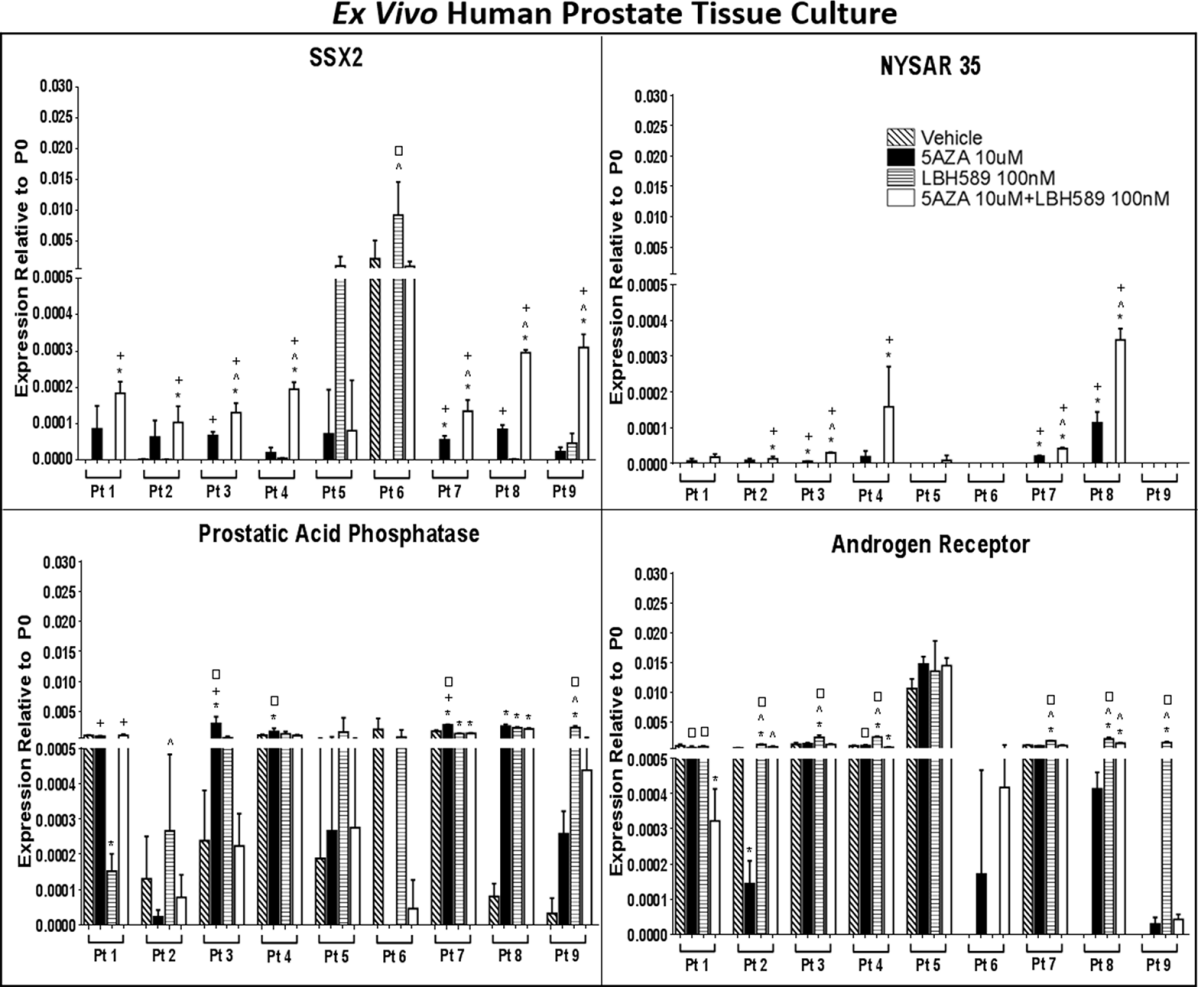
EMA treatment induces CTA expression in *ex vivo* human prostate tissue in a prostate biopsy drug culture model Quantitative analysis of relative expression of SSX2, NYSAR35, prostatic acid phosphatase (PAP) and androgen receptor (AR) mRNA in PC tissue biopsies treated with EMAs. RNA was evaluated by qRT-PCR for expression relative to an internal control transcript (P0) following EMA treatment. qRT-PCR was performed using primers specific for each gene and was conducted in triplicate. Error bars represent the mean and SD. A missing bar indicates that there was no detectable signal. Comparison between groups was made with a 1-way ANOVA followed by post hoc analysis with the Tukey test. *P* < 0.05 compared with vehicle (*), 5AZA 10 μM (^), LBH589 100 nM (+), or 5AZA 10 μM + LBH589 100 nM (□) treatment.

In order to determine if epigenetic treatments may enhance CTA expression in a more clinically relevant model, we utilized a novel *ex vivo* prostate tumor culture system (Figure 4) to treat tumor biopsies collected from 9 patients undergoing radical prostatectomy (Figure 5). To validate this model, we measured the expression levels of AR and PAP which are the targets of other vaccine therapies [48]. Baseline PAP expression was detectable in all 9 patient samples and 7 out of 9 samples had detectable expression of AR (*bottom row, right panel*). In concordance with *in vitro* studies (Supplementary Figure S6), EMAs had a limited impact on AR expression in this model though a subset of patient samples showed variable induction and repression of the AR pathway. In 7 patients, epigenetic treatment enhanced PAP expression in prostate tumor tissue following at least one treatment condition (*bottom row, left panel*). Interestingly, LBH589 alone increased PAP in 3 samples, similar to that observed evaluating PSA in the Phase I clinical trial with this agent in men with PC [49].

SSX2 mRNA was only detected in 1 out of 9 patients' tumor tissue cultures at baseline (Figure 5, *top row, left panel*). However, epigenetic treatments induced expression of SSX2 mRNA in all primary tumor tissue cultures. 5AZA alone and LBH589 alone significantly increased SSX2 levels in three and two patients samples, respectively. Combination of 5AZA and LBH589 treatment resulted in robust expression of SSX2 in 8 patient samples. NYSAR35 was not detected in any prostate tumor tissue cultures at baseline (*top row, right panel*). Three out of five prostate tumor cultures responded to 5AZA alone while LBH589 alone did not have any effect on NYSAR35 expression. Five patient tumor samples showed a synergistic impact on NYSAR35 expression following combinatorial treatment with 5AZA and LBH589. Taken together, our results indicate, that epigenetic treatment has the potential to increase CTA expression levels in tumor lesions *in situ* in human PC.

### Promoter methylation in SSX2 expression

We observed that SSX2 expression is considerably variable across PC cell lines with relatively high expression in LNCaP and 22rv1 cells and very low or undetectable expression in all others. To see if differences in baseline expression and responsiveness to treatment is reflected in methylation levels of the SSX2 promoter, we first conducted bisulfite genomic sequencing of promoter CpG islands of untreated DU145, LNCaP and 22rv1 cells (Supplementary Figure S7.). We analyzed two CpG islands separately, one from +940 to +664 and the other from +133 to −51, both relative to the transcriptional start site. Among the three cell lines analyzed, DU145 showed the highest methylation levels that correlated with near absent gene expression. However, methylation levels did not clearly associate with SSX2 expression levels in the LNCaP and 22rv1 cell lines. Similarly, methylation analysis of these cell lines at the +133 to −51 loci following treatment with 5AZA and/or LBH589 treatments did not reveal significant alterations, suggesting promoter methylation at these loci is not the sole factor controlling expression of SSX2.

### CTA as a potential biomarker for systemic disease

**Figure 6:**
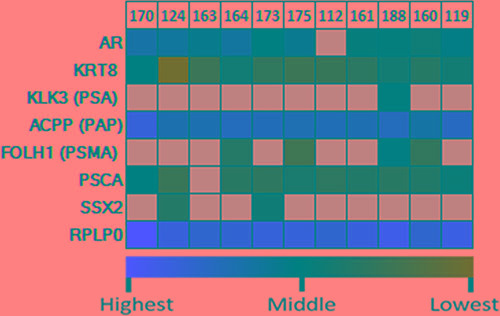
Quantitative analysis of expression of SSX2 and prostate-specific marker mRNA in circulating tumor cells qRT-PCR was performed using primers specific for each gene, and the P0 housekeeping gene is included for reference. Expression was color scaled from red (highest) to black (middle) to green (lowest). Grey indicates that no relevant expression was detected.

Next, we obtained peripheral blood samples from 11 patients diagnosed with PC (with variable disease status). EpCAM^+^ cells were isolated from CD45^−^ enriched buffy coats. We performed a qPCR microarray on the enriched CTCs, which showed a strong baseline expression of prostate specific markers, including PAP in all and AR and prostate stem cell antigen (PSCA) in 10 out of 11 patients (Figure 6). Further prostate specific markers were analyzed including PSA and prostate specific membrane antigen (PSMA). PSA was only detected in one patient while PSMA was expressed in 4 out of 11 CTC samples. Cytokeratin 8 was used to confirm tumor cells present in the isolates. CTCs from 2 out of 11 patients had detectable levels of SSX2 mRNA. In conclusion, SSX2 may be expressed in PC both *in situ* in tumor tissue and in circulating tumor cells.

## DISCUSSION

Recent advances in immune therapies have improved survival for patients with advanced PC, though with limited success to date. One major limitation of these agents is the lack of immunogenic, non-tolerizing target antigens expressed by tumor cells. CTAs have been identified as promising TAAs due to their restricted expression and immunogenicity [50–52]. Previous studies have identified heterogeneous expression of CTAs in a wide variety of cancers, including primary PC [52–54]. In this study, we tested the hypothesis that EMAs can induce expression of immunologically potentially relevant CTAs in human PC. We detected expression of 19 of the 29 tested CTAs across five PC cell lines with relatively high expression of multiple MAGE-A family members, SSX2, NY-ESO1, GAGE-2, XAGE-1, LIP1 and SPA17. An analytic review of Gene Expression Omnibus Data (GEOD) on urological malignancies by Kulkarni et al. [54] also showed a heterogenous panel of CTAs expressed in prostate cancer, with numerous MAGE-A and MAGE-B CTAs detected, similarly to a recent study by Maxfield et al. which analyzed the LNCaP cell line [16]. SSX family members were also detected with SSX2 and SSX4 (not analyzed in this report) being identified in the Kulkarni review [54] but not detected in LNCaPs in the Maxfield study [16]. Similar to our findings, SSX1, NXF-2 and SPANXC were also found in PC by this analysis. However, ADAM2, SSX5, PAGE1, TPX-1 (CRISP2), PRM2 (MAD-CT1) were detectable in PC in this report and not detected at baseline among our model cell lines suggesting considerable heterogeneity in the CTA repertoire within PC. NY-ESO1, NY-SAR35, XAGE-1, GAGE2 and PAGE5 were all detected in at least 1 cell line in this study but not reported in the above GEOD analysis [16, 54]. This is the first report of inducible expression of PAGE5 which has previously been detected at low levels in 3 of 12 prostate carcinoma samples in the Human Protein Atlas [55] (http://www.proteinatlas.org/ENSG00000158639-PAGE5/cancer/tissue/prostate+cancer) and trace amounts of PAGE5 was also detected in the PC3 cell line (http://www.proteinatlas.org/ENSG00000158639-PAGE5/cell).

In comparison, the LNCaP, LAPC4 and 22rv1 cell lines expressed a larger repertoire of CTAs, including at least 8 antigens each while PC3 and DU145 cells expressed a smaller palette of CTAs. Similar complexity was also reflected in the study of Maxfield et al. on the LNCaP cell line [16]. This heterogeneity is not surprising given prior studies evaluating CTA expression in clinical samples. For example, CTA expression levels and patterns highly correlate with advanced disease [22, 56], with microarray studies identifying significant differences in SSX2 expression between primary and metastatic PC lesions. A serum IgG screening study of patients with PC [36] detected a variety of antibodies specific to CTAs including NY-ESO-1, LAGE-1, NXF-2 and SSX-2, suggesting that a variety of CTAs are expressed on PC cells. Induction of CTA-specific immune response limits tumor growth as demonstrated in preclinical studies [57] and have shown some promising clinical benefits in various tumor models [58, 59].

The heterogeneous expression of CTAs theoretically limits the utility of this class of TAAs due to absent or aberrant expression in patient tumors. Multiple studies on genome-wide epigenetic analysis have demonstrated that silencing of CTAs is associated with tumor pathogenesis and that there is hypermethylation of CTA promoter regions in multiple cancer types [28–31]. Chemical reversal of epigenetic silencing through the use of EMAs has been shown in melanoma to induce CTA expression, restore immune recognition and promote tumor clearance [25, 28, 29, 35, 36, 60–62]. Similarly, we identified this phenomenon in PC using hypomethylating agents alone or in combination with an HDAC inhibitor. Both additive and synergistic increases in CTA expression occurred in all cell lines following treatment with both EMAs, inducing homogenous expression patterns in CTAs across all PC cell lines. Furthermore, our results suggest promoter methylation is not the sole mechanism by which EMAs induce CTA expression. Previous studies suggest that epigenetic regulation of certain genes may be the result of core histone modification that results in the increase of spatial availability for transcription [63]. Gene expression may also be the result of demethylation of regions outside of the promoter that were not analyzed in our study. We observed that AR-expressing cell lines responded less to epigenetic treatment while androgen-independent cell lines were more inducible. This finding is in accordance with those of Suyama et al. [56], who reported that MAGE-A2 expression was rescued in non-expressing PC3 and DU145 cell lines with a remarkably high fold-change over vehicle while LNCaP and CWR22 were not inducible by 5AZA.

To evaluate the translational relevance of these findings, we developed a novel approach to test EMAs in human cancer with an *ex vivo* tissue culture with human PC specimens. Similar drug cultures were used by Centenera et al. [64] to test biological responses to novel HSP90 inhibitors in PC. This study observed biological effects of these drugs not yet observed in preceding experiments done on cell lines and in animal models suggesting the value of *ex vivo* drug cultures as a potentially more appropriate preclinical model for rational selection of therapeutic agents. The absence of CTA expression in vehicle treated samples matches previously published work in tissue microarrays. Similar to the *in vitro* results, EMAs induced expression of SSX2 and NYSAR35 in primary human PC tissue culture. This pattern of responses identifies anticipated heterogeneity in primary human PC that can be modified through the use of EMAs. These results did not address the durability of this response or the optimal frequency of treatment to induce alterations in gene expression and is the subject of ongoing experiments. This model is also well suited to screen novel EMAs in development for synergistic alterations in CTA expression. These results identify a strategy in which combinatorial epigenetic treatments can induce a large palette of tumor-selective neoantigens as part of a multifaceted immunotherapeutic strategy to treat PC. Multi-epitope immune targeting therapy of prostate-specific epitopes have been studied and shown efficient at boosting cellular immune responses in multiple studies in PC [65]. Additionally, antigen spreading after efficient single-epitope vaccination correlating with improved overall survival has been also shown in PC [66]. The induction of polyclonal anti-tumor response against multiple CTAs on prostate tumor cells may promote more efficient effector mechanisms, higher intensity of overall immune responses and more successful tumor targeting. A Phase I clinical trial (NCT106158) enrolling patients with NY-ESO-1 expressing tumors including 8 advanced esophageal and 2 PC patients immunized with recombinant NY-ESO-1 protein found a heteroclitic immune response to 10 CTAs in addition to NY-ESO-1 protein following immunization [51]. Although the limited number of patients did not allow the investigators to establish clear correlation between CTA-specific immune responses and clinical outcomes in that study, other studies with the same NY-ESO-1 recombinant protein vaccine enrolling patients with Stage IV esophageal, Stage D3 PC, or Stage IV melanoma detected both antibody, CD4 an CD8 T cell responses against the vaccine protein, which associated with beneficial clinical effects such as tumor regression or stable disease for a prolonged time in six of seven evaluable patients. PSA levels temporarily stabilized in 3 of 4 PC patients with recurrent hormone-refractory tumors [58]. Additionally, vaccine studies utilizing CTAs [59] and other tumor-associated targets have suggested the association of clinical response with the detection of multi-epitope cellular and humoral immune responses against antigens other than the original vaccine epitope [67]. These results suggest that biological doses of EMAs may result in expression of a large palette of CTAs in PC cells and potentially synergize with other immune therapies to enhance tumor lysis.

These results support a rationale to utilize currently available vaccines targeting CTAs in combination with EMAs. However, the timing and appropriate dose of EMAs in this context is unknown. This is even more critical of an issue with this class of drugs as clinical benefit from hypomethylating agents was not observed until these agents were administered below the maximally tolerated dose. The potential for biomarkers using CTCs may allow real-time monitoring of gene expression alterations to identify an optimal biologic dose for this specific context of use. We demonstrate proof-in-concept data identifying expression of SSX2 in EpCAM positive circulating tumor cells from patients with advanced PC. Gene expression analysis identified expression of multiple PC specific genes demonstrating the specificity of this assay and is consistent with prior studies showing preferential expression of SSX2 in metastatic lesions [22]. SSX2 has been previously proposed as a promising vaccine target in PC [52, 57] and is being evaluated as part of a combination immunotherapy regimen in a Phase I clinical trial (NCT02625857) for men with CRPC. Our finding is in accordance with a recent report by Bloom et al. detecting SSX2 mRNA in 19 of 54 (35%) of CD45−/EpCAM+/CD63+ circulating tumor cell samples in PC patients [68]. Our detection was lower at 2 out of 11 (18%). Our study has also been sampling a narrower group, patients with advanced PC while the study of Bloom et al. has analyzed PC patients from a broad spectrum of disease from newly diagnosed through CRPC. These studies do not evaluate other EpCAM-negative events, which may exhibit higher SSX2 expression as this gene has been associated with epithelial-mesenchymal transitions [68] that may have been missed in this study. The extent to which gene expression alterations occur in CTCs relative to the primary tumor is unknown at this time but is an easily testable hypothesis through the integration of paired tumor and blood analytics. Validation of these assays in prospective clinical trials may further speed the development of these combination strategies as well as improve patient stratification for these treatments.

EMAs have become the focus of renewed interest for development of cancer therapies as preclinical data has demonstrated potent and wide-ranging anti-tumor effects when administered in low, biological doses. This study has demonstrated that PC cells are potent expressors of novel immunogenic targets in human PC and that EMA treatments enhance CTA expression and even restore it in epigenetically silenced androgen-independent PC cells. This suggests that EMAs may offer a mechanism to induce tumor-selective CTL epitopes and may promote more efficient targeting by induction of a multi-epitope immune response. Exploring if EMAs may enhance CTA-specific CTL responses and tumor clearance is an important question to address to evaluate potential future clinical significance.

## MATERIALS AND METHODS

### Cell lines and cell culture

All cell lines except LAPC4 were maintained in RPMI1640 supplemented with 10% FBS, 0.1% beta-mercaptoethanol, 1% sodium Pyruvate, 2% penicillin-streptomycin, and 1% essential amino acids. LAPC4 cells were grown in DMEM medium supplemented with 0.5% beta-mercaptoethanol, 1% sodium Pyruvate, 2% penicillin-streptomycin, and 20% FBS, (Gibco, Life Technologies) in Poly-D-Lysin coated tissue culture dishes (Corning).

### Primary *ex vivo* PC tissue culture

Human PC tissues were obtained from patients undergoing radical prostatectomy at the University of Wisconsin-Madison. All patients were consented under an Institutional Review Board (IRB) protocol #20130653. Each core was cut into ~1 mm^3^ pieces with a sterile scalpel and scissors. Absorbable gelatin sponges (Ethicon) were cut into pieces to fit in a 24-well tissue culture plate (Figure 4). Sponges were soaked in Ham's F-12 media (Mediatech) supplemented with 0.25 units/ml regular insulin (Sigma-Aldrich), 1 μg/mL hydrocortisone (Sigma), 5 μg/mL human transferrin (Sigma), 2.7 mg/ml dextrose (Sigma), 0.1 nM non-essential amino acids (Hyclone), 100 units/ml and 100 μg/mL Penicillin/Streptomycin respectively (Mediatech), 2 mM L-glutamine (Sigma), 25 μg/mL bovine pituitary extract (Invitrogen) 1% FBS (Gibco, Life Technologies) until fully saturated. Tissue was placed on the sponges and cultured for up to 6 days at 37C at 5% CO_2_ and 0.5 ml. Media was replaced, daily.

### Reagents

5-Aza-2′-deoxycytidine (5AZA) was purchased from Sigma-Aldrich (St. Louis, MO), and Panobinostat (LBH589) was purchased from Selleckchem (Houston, TX). Reagents were dissolved and stored in DMSO freezer aliquots.

### Epigenetic drug treatments of cell lines and human primary tissue cultures

Cells and primary tissue cultures were treated with 10 μM 5AZA or DMSO every 24 hours for 72 hours with media replacements. 10 or 100 nM LBH589 was added for the last 24 hours. All cells and tissue were harvested at 72 hours. Genomic DNA was extracted with the DNeasy blood and tissue kit (Qiagen). Total RNA was isolated with RNeasy mini kit (Qiagen) from cells and the Aarum Fatty and Fibrous Tissue Kit (Bio-Rad) from prostate tissue.

### Expression analysis by quantitative PCR and data interpretation

Total RNA was quantified by a NanoDrop 1000 spectrophotometer and 1μg total RNA was reverse transcribed using the iScript cDNA synthesis kit (Bio-Rad). 1 μL of the cDNA synthesis reaction was used to perform qRT-PCR using the Sso advanced universal SYBR green supermix (Bio-Rad) according to the manufacturer's protocol. Reactions were run on a CFX Connect (Bio-Rad) Real-Time PCR machine. CTA expression relative to the P0 control gene [69] as analyzed using the 2^-ΔCt^ method (http://nar.oxfordjournals.org/content/29/9/e45.long). P0 forward: 5′-GACAATGGCAGCATCTACAAC-3′; P0 reverse: 5′-GCAGACAGACACTGGCAAC-3′. CTA primer sequences are included in Table 1 [70].

qPCR data sets from *in vitro* assays are presented in both heat maps (main Figures 1–3) and on bar graphs (in Supplementary Figures S1–S5) to provide alternative ways to analyze patterns in both gene expression and inducibility in a relatively large data set. Due to complexity, statistical analysis is depicted on bar graphs only (Supplementary Figures S1–S5). To better illustrate inducibility for each individual gene, the color scales on the heat maps were normalized within each row, across all cell lines. Therefore, gene expression levels between different genes may not represent the same color on the color scale on the heat map. Bar graphs provide a comparable measure to show differences in absolute expression levels between the various CTAs measured.

### Bisulfite genomic sequencing

Genomic DNA was bisulfite converted using the EpiTect bisulfite kit (Qiagen) and amplified in 50 μL PCR reactions containing 2 U ZymoTaq DNA polymerase (Zymo Research), 1x reaction buffer (Zymo Research), 250 μM each dNTPs, 1 μM forward primer, 1 μM reverse primer, and 50ng bisulfite converted DNA. Three primer pairs specific for the SSX2 promoter were used: forward 1: 5′-GGGTAGGGTGGTGTATGTTTGT-3′; reverse 1: 5′-ACCTTAACCAATCCTCCAACCT-3′; forward 2: 5′-GGAAGGATTTTTTGAGTTTAGGA-3′; reverse 2: 5′-TCTACCTTAACCAATCCTCCAA-3′; forward 3: 5′-AAGGATGATGGATTAATTAGGGT-3′; reverse 3: 5′-AATCCAAAAAAAAAATCAAACC-3′. Cycling conditions were as follows: 95°C for 10 min, 40 cycles of 95°C for 30 sec, appropriate annealing temperature for 30 sec, 72°C for 1 min, and a final extension step of 72°C for 7 min. PCR products were purified using either the QIAquick PCR purification kit (Qiagen) or the QIAquick gel extraction kit (Qiagen). Purified products were then cloned into the pGEM-T easy vector (Promega), grown on AMP/IPTG/X-gal plates, cultured in suspension overnight, collected using the QIAprep spin miniprep kit, and sent to the UW-Madison Biotechnology Center for standard Sanger sequencing.

### Patient CTC mRNA analysis

### VERSA capture and CTC mRNA extraction

The VERSA-based capture of circulating tumor cells from peripheral blood samples followed by mRNA extraction was previously validated and reported by Sperger et al. [71]. The VERSA device [71–74] was injection-molded by Proto Labs (USA). Blood samples were collected from patients consented under the University of Wisconsin-Madison IRB protocol #XP08813. Blood was collected into K2EDTA Vacutainer tubes (BD Biosciences, USA) and PBMCs were isolated on Ficoll-Paque (GE, USA) gradient. PBMCs were then subjected to CD45 depletion following manufacturer's protocol (Dynabeads, Life Tech, USA) and the CD45^−^fraction was incubated with paramagnetic particles (PMPs) (Dynabeads^®^ FlowComp^™^ Flexi kit, Life Technologies, USA) coated with EpCAM antibody (R&D Systems, USA). The PMP-bound cells were then captured in the VERSA device.

### TaqMan^®^ reverse transcription polymerase chain reaction

The mRNA elution sample was reverse transcribed using a High Capacity cDNA Reverse Transcriptase kit (Life Tech, USA), according to manufacturer's directions using Bio-Rad C1000 Thermo Cycler (Bio-Rad, USA). The RT reaction (12.5 μL) was then amplified for 10 cycles using TaqMan^®^ PreAmp (Life Tech, USA) according to manufacturer's directions and diluted 1:5 in 1× TE (10 mM Tris-HCL pH8, 1 mM EDTA). For TaqMan^®^ assays, 5 μL of diluted cDNA template was mixed with 10 μL iTaq® master mix (Bio-Rad, USA), 1 μL TaqMan^®^ Gene Expression Assay (Specified in Table 1, Life Technologies, USA) and 4 μL nuclease free (NF) water. Each reaction was amplified for 40 cycles (denatured at 95°C for 15 seconds followed by annealing at 60°C for 1 minute) using a CFX Connect^®^ Real-Time PCR System (Biorad, USA).

### Flow cytometry analysis of SSX expression

SSX expression was analyzed by intracellular staining using a goat polyclonal SSX antibody (Clone N-18, Santa Cruz). The antibody was conjugated to Alexa^®^ 568 followed by manufacturer's protocol (Molecular Probes, Invitrogen). Cells were first stained with violet 510 fixable Live/Dead stain (Tonbo Biosciences) followed by fixation, permeabilization (FoxP3 Transcription Factor kit, eBioscience) and intracellular staining following the manufacturer's protocol. Samples were acquired on an LSR Fortessa (BD Biosciences) instrument and data analyzed by the FlowJo software v9.9 (Treestar, OR). Mean Fluorescent Intensity was analyzed on gated live, single cells.

### Statistical analysis

Comparison between groups was made with a 1-way ANOVA followed by post hoc analysis with the Tukey test for correction of multiple testing.

## SUPPLEMENTARY MATERIALS FIGURES AND TABLES


