# Supplementary material for: Inducible expression of cancer-testis antigens in human prostate cancer
Source: Oncotarget. 2016 Oct 17;7(51):84359–74. doi: 10.18632/oncotarget.12711 (PMC5341296; doi:10.18632/oncotarget.12711)
Supplement: Supplementary file 1 [file oncotarget-07-84359-s001.pdf]

Inducible expression of cancer-testis antigens in human prostate cancer

Supplementary Materials

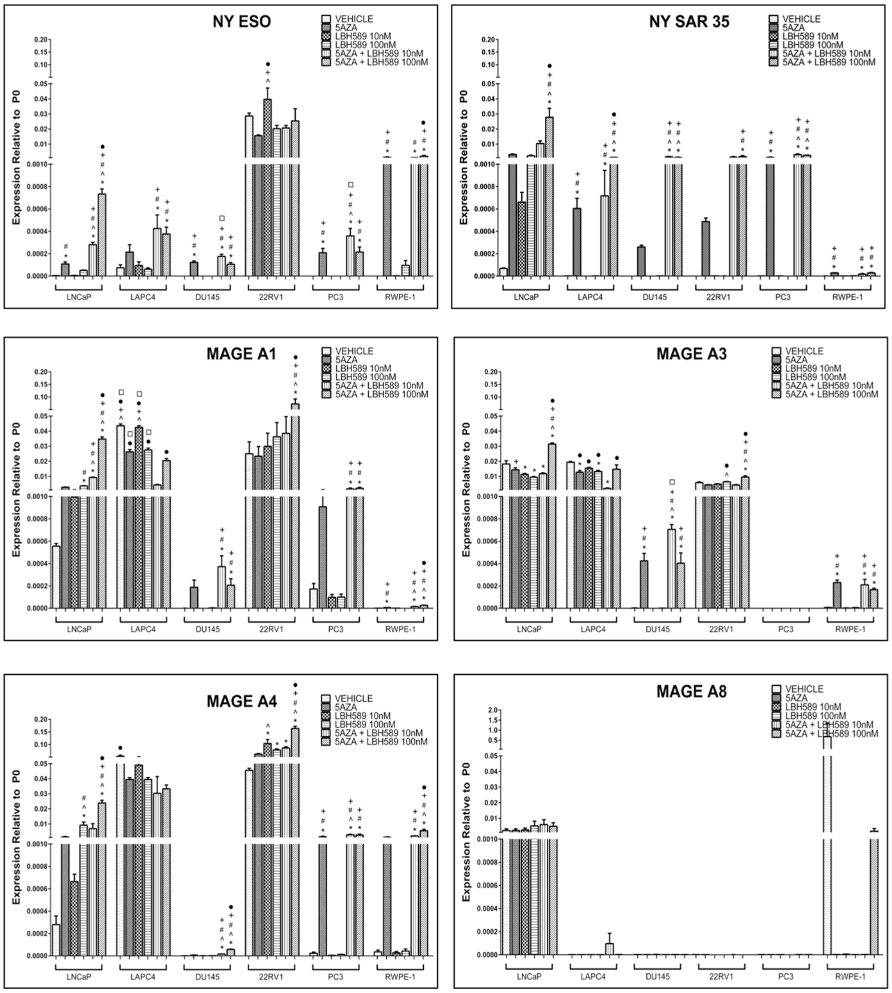

Supplementary Figure S1: Quantitative analysis of relative expression of NY-ESO, NY-SAR-35, MAGE-A1, MAGE-A3, MAGE-A4, MAGE-A8 and mRNA in PC cell lines treated with EMAs. RNA was evaluated by qRT-PCR for expression relative to an internal control transcript (P0) following EMA treatment. qRT-PCR was performed using primers specific for each gene, conducted in triplicate, and repeated in an independent experiment. Error bars represent the mean and SD. Comparison between groups was made with a 1-way ANOVA followed by post hoc analysis with the Tukey test. $P < 0.05$ compared with vehicle (*), 5AZA 10 µM (^), LBH589 10 nM (#), LBH589 100 nM (+), 5AZA 10 µM + LBH589 10 nM (●), or 5AZA 10 µM + LBH589 100 nM (□) treatment.

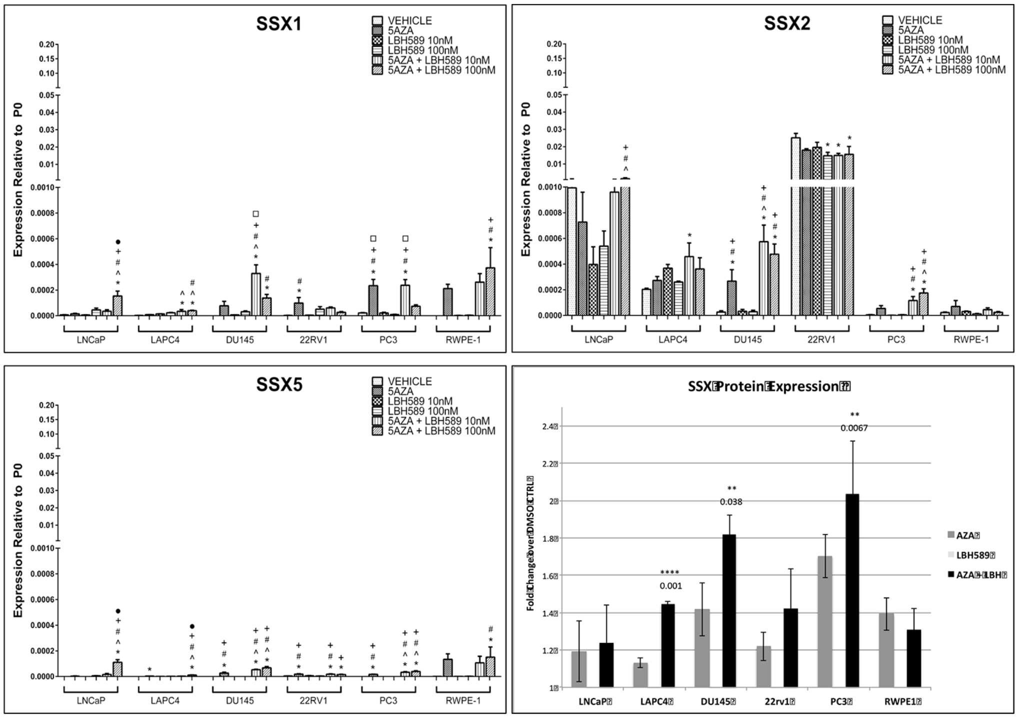

Supplementary Figure S2: Quantitative analysis of relative expression of SSX1 (top row left), SSX2 (=) and SSX5 (*bottom row left*) mRNA in PC cell lines treated with EMAs. RNA was evaluated by qRT-PCR for expression relative to an internal control transcript (P0) following EMA treatment. qRT-PCR was performed using primers specific for each gene, conducted in triplicate, and repeated in an independent experiment. Error bars represent the mean and SD of 6 wells from these 2 experiments. Comparison between groups was made with a 1-way ANOVA followed by post hoc analysis with the Tukey test. $P < 0.05$ compared with vehicle (*), 5AZA 10 μM (^), LBH589 10 nM (#), LBH589 100 nM (+), 5AZA 10 μM + LBH589 10 nM (●), or 5AZA 10 μM + LBH589 100 nM (□) treatment. Bottom Row Right Panel. Flow Cytometry analysis of SSX protein expression after EMA treatment. Cells were treated with SSX expression (SSX1-9) was detected by flow cytometry with a polyclonal SSX antibody (SC N-18). Data represent fold change of Mean Fluorescent Intensity over DMSO control. Statistical analysis was performed by 1-way ANOVA followed by the Tukey post-hoc test. Data represent 2–3 independent experiments. Comparison between groups was made with a 1-way ANOVA followed by post hoc analysis with the Tukey test. $P < 0.05$ compared with vehicle (*), 5AZA 10 μM (^), LBH589 10 nM (#) treatment.

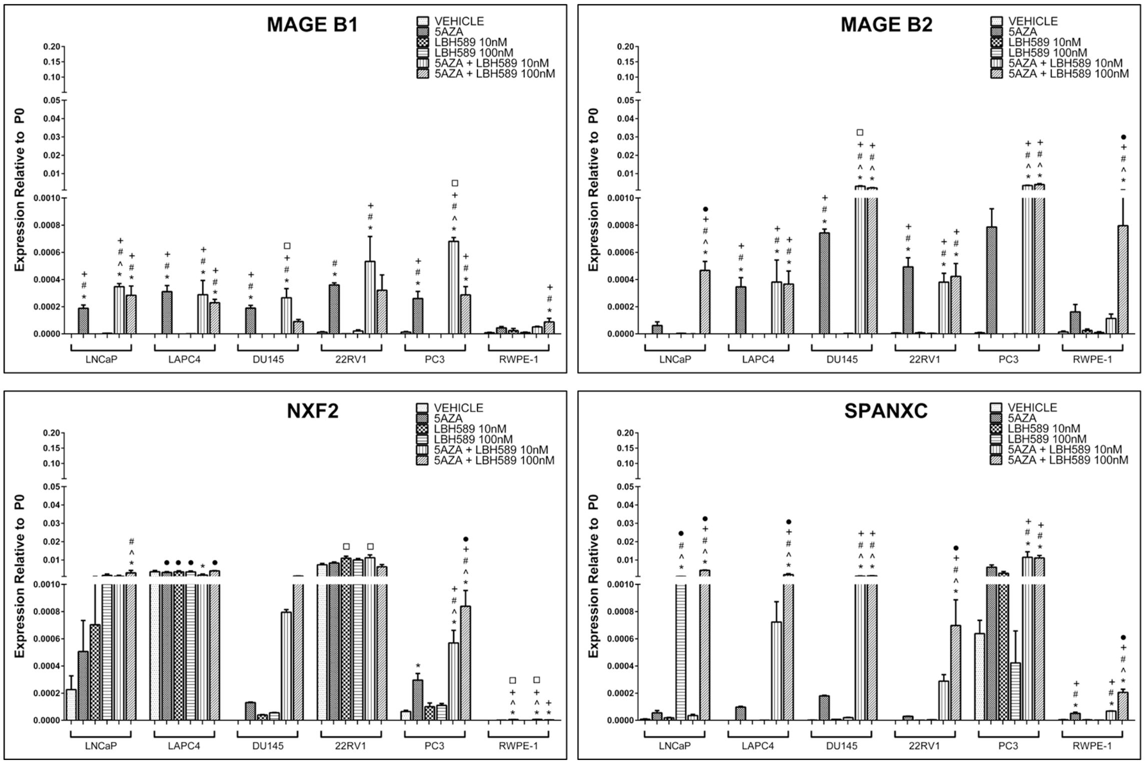

Supplementary Figure S3: Quantitative analysis of relative expression of MAGEB1, and MAGEB2, SPNAXC and NXF2 mRNA in PC cell lines treated with EMAs. RNA was evaluated by qRT-PCR for expression relative to an internal control transcript (P0) following EMA treatment. qRT-PCR was performed using primers specific for each gene, conducted in triplicate, and repeated in an independent experiment. Error bars represent the mean and SD of 6 wells from these 2 experiments. Comparison between groups was made with a 1-way ANOVA followed by post hoc analysis with the Tukey test. $P < 0.05$ compared with vehicle (*), 5AZA 10 µM (^), LBH589 10 nM (#), LBH589 100 nM (+), 5AZA 10µM + LBH589 10 nM (●), or 5AZA 10 µM + LBH589 100 nM (□) treatment.

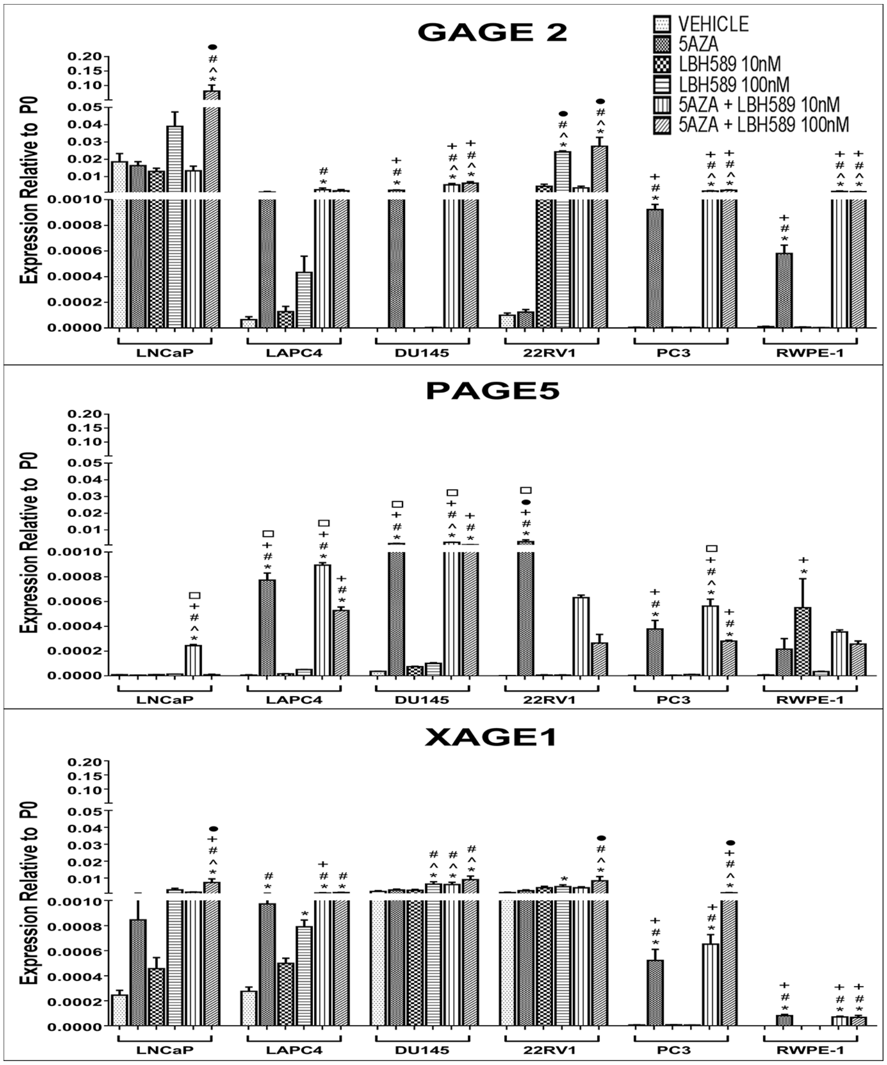

Supplementary Figure S4: Quantitative analysis of relative expression of GAGE2, PAGE5, and XAGE1 mRNA in PC cell lines treated with EMAs. RNA was evaluated by qRT-PCR for expression relative to an internal control transcript (P0) following EMA treatment. qRT-PCR was performed using primers specific for each gene, conducted in triplicate, and repeated in an independent experiment. Error bars represent the mean and SD of 6 wells from these 2 experiments. Comparison between groups was made with a 1-way ANOVA followed by post hoc analysis with the Tukey test. $P < 0.05$ compared with vehicle (*), 5AZA 10 μM (^), LBH589 10 nM (#), LBH589 100 nM (+), 5AZA 10 μM + LBH589 10 nM (●), or 5AZA 10 μM + LBH589 100 nM (□) treatment.

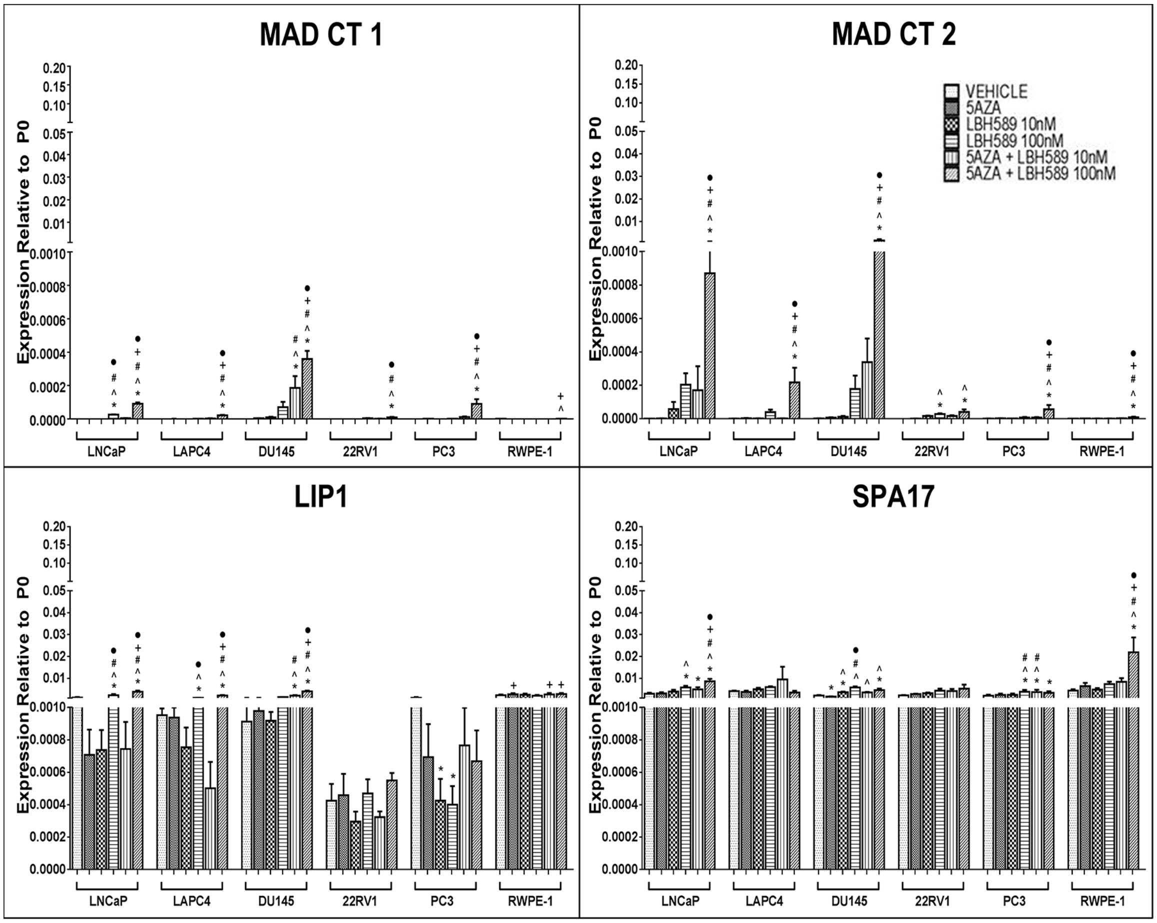

Supplementary Figure S5: Quantitative analysis of relative expression of non CT-X antigen (MAD CT1, MAD CT2, LIP1, SPA17) mRNA in PC cell lines treated with EMAs. RNA was evaluated by qRT-PCR for expression relative to an internal control transcript (P0) following EMA treatment. qRT-PCR was performed using primers specific for each gene, conducted in triplicate, and repeated in an independent experiment. Error bars represent the mean and SD of 6 wells from these 2 experiments. Comparison between groups was made with a 1-way ANOVA followed by post hoc analysis with the Tukey test. $P < 0.05$ compared with vehicle (*), 5AZA 10 µM (^), LBH589 10 nM (#), LBH589 100 nM (+), 5AZA 10 µM + LBH589 10 nM (●), or 5AZA 10 µM + LBH589 100 nM (□) treatment.

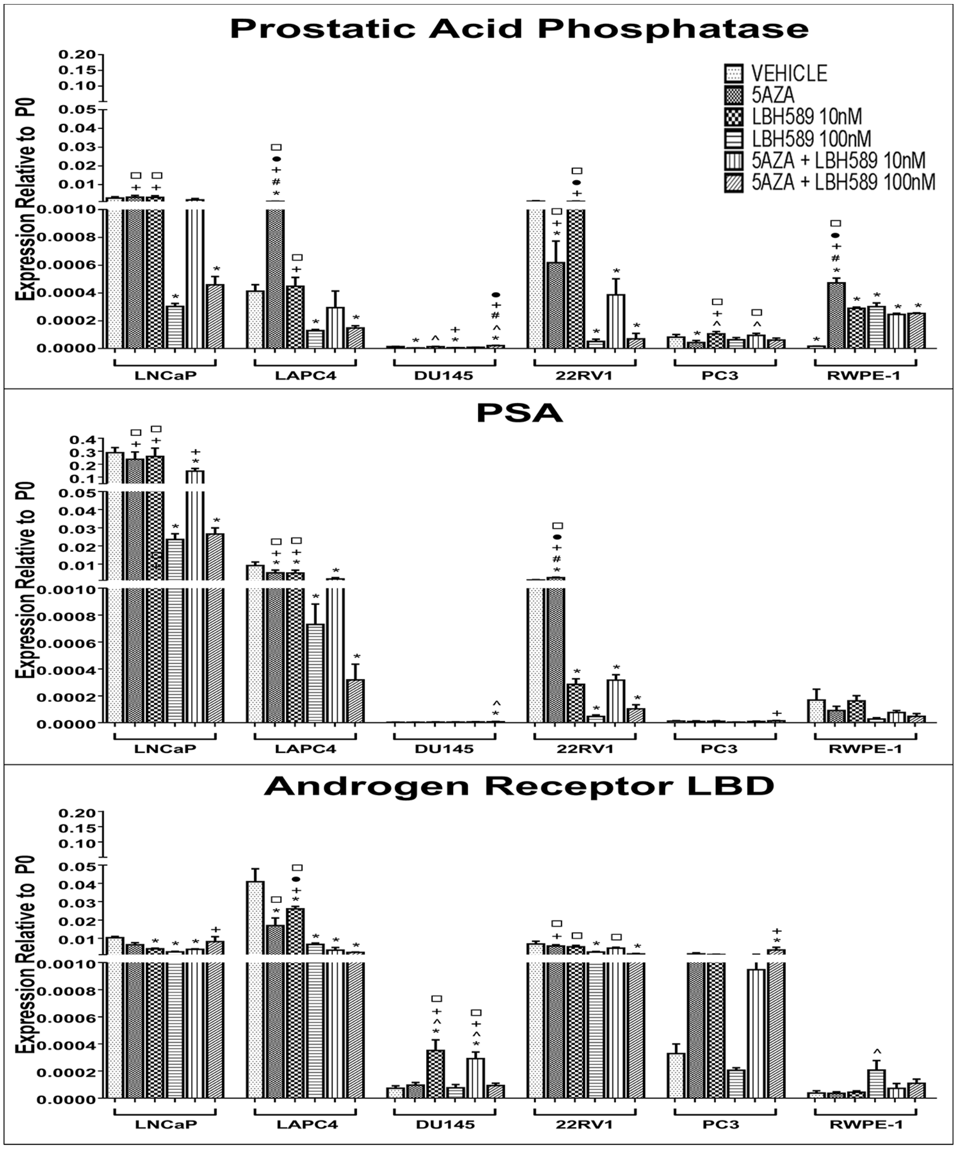

Supplementary Figure S6: Quantitative analysis of relative expression of PAP, PSA, and AR mRNA in PC cell lines treated with EMAs. RNA was evaluated by qRT-PCR for expression relative to an internal control transcript (P0) following EMA treatment. qRT-PCR was performed using primers specific for each gene, conducted in triplicate, and repeated in an independent experiment. Error bars represent the mean and SD of 6 wells from these 2 experiments. Comparison between groups was made with a 1-way ANOVA followed by post hoc analysis with the Tukey test. $P < 0.05$ compared with vehicle (*), 5AZA 10 µM (^), LBH589 10 nM (#), LBH589 100 nM (+), 5AZA 10 µM + LBH589 10 nM (●), or 5AZA 10 µM + LBH589 100 nM (□) treatment.

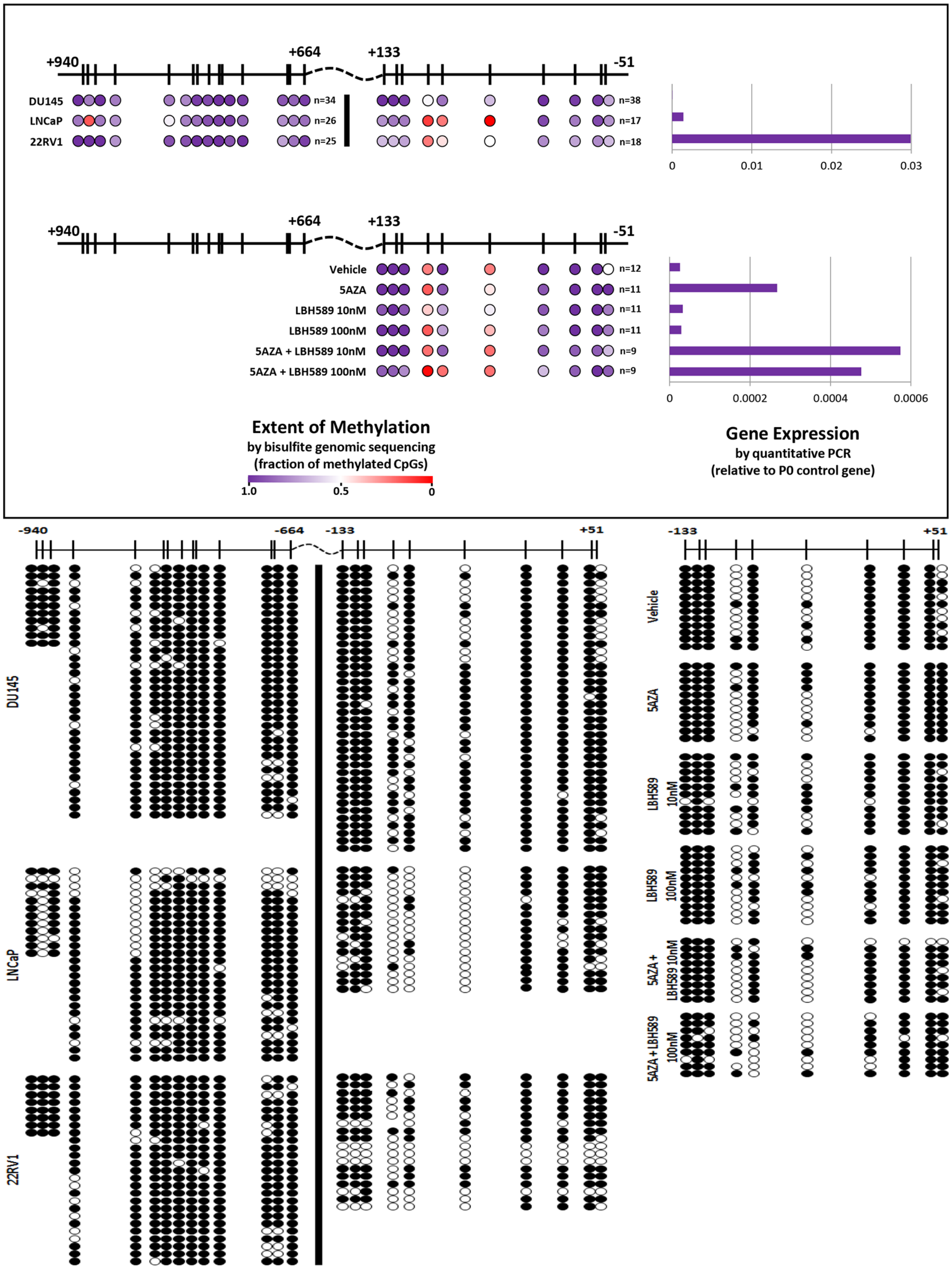

Supplementary Figure S7: Panel A. Bisulfite genomic sequencing of the SSX2 gene promoter at baseline in DU145, LNCaP, and 22RV1 cell lines and after treatment with 5AZA and/or LBH589 in DU145 cells. Two CpG islands were analyzed separately, one from +940 to +664 and the other from +133 and −51, both relative to the transcriptional start site. The position of each CpG site is indicated by a vertical hash mark. Multiple clones (number shown to the right of each group) were obtained, each representing a unique DNA sequence from the cell culture. The fraction of methylated CpG sites was color scaled from purple (all sequences methylated at that CpG site) to red (no sequences methylated at that CpG site). Two CpG islands were analyzed separately, one from +940 to +664 and the other from +133 and −51, both relative to the transcriptional start site. Bar graph on right top represents SSX2 gene expression relative to P0 at baseline in DU145, LNCaP, and 22RV1 cell lines and bar graph on right bottom shows SSX2 gene expression relative to P0 at baseline after treatment with 5AZA and/or LBH589 in DU145 cells. Panel B. Figures showing raw methylation data for each individual clone are included. Each row represents one clone. A black circle represents a methylated CpG site while a white circle represents an unmethylated CpG site. The left column shows baseline methylation in multiple clones in the DU145, LNCaP, and 22RV1 cell lines and the right column shows DU145 cells after treatment with vehicle, 5AZA and/or LBH589.
